# Amphibian supercooling capacity is not limited to sub-zero thermal environments

**DOI:** 10.1038/s41598-025-24105-5

**Published:** 2025-11-17

**Authors:** Philippe J. R. Kok, Bruno B. Wisse, Marlena Kapuściak, Margarita Lampo

**Affiliations:** 1 Department of Ecology and Vertebrate Zoology, University of Lodz, Banacha Str. 12/16, 90-237 Łódź, Poland; 2 Institute for Biodiversity and Ecosystem Dynamics, University of Amsterdam, 1098 XH Amsterdam, The Netherlands; 3 Instituto Venezolano de Investigaciones Científicas, Miranda, Venezuela; 4 Fundación para el Desarrollo de las Ciencias Físicas, Matemáticas y Naturales, Caracas, Venezuela

**Keywords:** Cold hardiness, Evolution, Physiological freeze avoidance, Skin microbiome, Tropical montane, Ecology, Ecology, Zoology

## Abstract

Freeze-tolerant amphibians initiate controlled freezing using ice nucleators and survive internal ice formation by accumulating cryoprotectants. In contrast, freeze-avoidant (supercooling) species rely on the inhibition of ice nucleators to prevent freezing altogether. All confirmed supercooling species are native to the Northern Hemisphere and regularly endure negative temperatures. The occurrence, ecological role, and underlying mechanisms of supercooling in amphibians remain poorly understood. Here, we demonstrate for the first time that amphibian supercooling capacity may be present even if not expressed (i.e., latent) and not limited to freezing thermal environments. Exploratory metagenomic data allow us to evaluate whether skin-associated bacteria could contribute to freeze avoidance. In addition, using field experiments, we assess cold and dehydration tolerance limits in two syntopic amphibian species from a high tepui summit (Roraima-tepui in Venezuela) and explore the potential role of cryoprotective dehydration in facilitating supercooling. Despite being syntopic, these species showed striking differences in thermal and dehydration tolerance. Physiological freeze avoidance in tropical montane amphibians is shown to be associated with low critical thermal minima, high dehydration tolerance and possibly antifreeze-producing skin microbiota, although the latter needs further investigation. These traits may determine species persistence under shifting climatic regimes, particularly in thermally variable montane systems.

**Supplementary Information:**

The online version contains supplementary material available at 10.1038/s41598-025-24105-5.

## Introduction

Ongoing climate change is reshaping thermal regimes worldwide, which particularly affects ectotherms such as amphibians^1^. The intensification of anthropogenic environmental stressors underscores the importance of identifying and characterising the physiological and ecological adaptations that enable ectotherms to survive in extreme environments. Such knowledge is essential for predicting species resilience and guiding conservation efforts for cold-adapted ectotherms globally. Physiological studies on freeze tolerance and freeze avoidance (= supercooling) are typically justified only when microhabitat temperature profiles demonstrate sustained sub-zero temperatures for periods long enough to induce partial tissue freezing. This approach might limit the recognition of new or convergent cold-adapted traits in tropical species from thermally buffered microhabitats.

Supercooling refers to the ability of some organisms to avoid freezing despite exposure to negative temperatures^2–5^. While supercooling, their body temperature (T_b_) substantially drops below the freezing point of water (0 °C) and the equilibrium freezing point of their body fluids (ca. -0.6 °C), without the formation of ice in body tissues^6^. This cold hardiness adaptation is one of the most remarkable survival strategies, and the study of supercooling in ectotherm vertebrates, such as amphibians, is pivotal to the understanding of both ecological physiology and cryobiology. Amphibians’ moist and permeable skin and the biophysical constraints of their high water volume inherently limit their ability to supercool compared to other terrestrial ectothermic taxa like insects or reptiles^3,6–10^.

Freeze-tolerant amphibians use ice nucleators (substances that promote the transition from liquid state to ice) to initiate controlled freezing and tolerate internal ice formation through the accumulation of high concentrations of cryoprotectants (e.g., glucose). In contrast, freeze-avoidant (supercooling) amphibians rely on the inhibition of ice nucleators to prevent freezing altogether^11–13^. Freeze tolerance and supercooling are strategies that are generally mutually exclusive since they involve antagonistic physiological and biochemical processes^11^. Historically, much more attention has been given to freeze-tolerant species from temperate regions (see reviews in, e.g.^6,11,14,15^), leaving supercooling species largely underrepresented, particularly in tropical montane environments where unexpected cold events may occur, or might have occurred during Quaternary climate oscillations. The prevalence and ecological significance of supercooling in amphibian species, particularly in high elevation tropical taxa, thus remain an open question.

Because supercooling is a transient state that can be challenging to observe and detect in field conditions, confirmed cases of supercooling in amphibians are scarce^13^. To the best of our knowledge, convincing evidence of freeze avoidance through supercooling has been primarily documented in the anurans *Anaxyrus cognatus* and *A. woodhousii*^15^, *Aquarana septentrionalis* and *Lithobates pipiens*^16^, *Spea bombifrons*^13^, *Anaxyrus americanus*^17^ and in the urodeles *Plethodon cinereus* and *Ambystoma laterale*^17^, all native to the Northern Hemisphere. All these species are routinely exposed to negative temperatures in their natural environment and die if temperature drops below their supercooling point (SCP; i.e., freeze). Because supercooling is essentially unstable over time, the longer an organism remains in a supercooled state, the greater the risk of (lethal) spontaneous ice nucleation. Therefore, this strategy is probably more developed in amphibians experiencing short-term cold exposures and modestly sub-zero conditions. As previously documented, the lowest SCP recorded in supercooling amphibians is ca. -4.30 °C^3^. Rodríguez et al.^18^ reported elevated glucose levels (essential for protecting intracellular structures during freezing) and a maximum SCP of -3.68 °C (mean -1.60 °C) in the Andean toad *Rhinella spinulosa*, without specifying whether these toads are freeze-tolerant or if the observed SCPs were lethal. Similarly, elevated glucose levels have recently been detected in some high-elevation Andean frogs in the genus *Pristimantis*, all shown to be freeze-tolerant, not supercooling^19^. Like their Northern Hemisphere counterparts, these tropical species are routinely exposed to negative temperatures in their environment.

Churchill and Storey^20^ argued that freeze tolerance likely derives from established physiological adaptations to water stress in amphibians. However, it is unclear if an elevated dehydration tolerance is a contributing factor for supercooling amphibians.

We recently initiated studies on the physiological and ecological adaptations of ectotherm vertebrates inhabiting climatically extreme and naturally fragmented paleoenvironments (i.e., characterised by prolonged climatic and geophysical isolation), such as tepui summits (Precambrian insular continental landscapes in northern South America). Our fieldwork notably revealed the outstanding ability to withstand cold and experimentally-induced freezing temperatures in *Oreophrynella quelchii*, a small toad living in a currently non-freezing environment. Our findings also highlighted the potential contribution of skin-associated bacteria to amphibian supercooling, which motivated the use of a metagenomic approach to profile and explore skin bacterial communities in *O. quelchii*. In this study, we present the scientific framework underlying these discoveries, assess the limits of cold and dehydration tolerance in *O. quelchii* and the syntopic *Pristimantis aureoventris*, and investigate the potential role of cryoprotective dehydration in facilitating supercooling. We also provide novel insights into the putative contribution of skin-associated bacteria to amphibian freeze avoidance, a promising new line of research.

## Materials and methods

### Study site and experimental species

All experiments were performed in our field laboratory located on the summit of Roraima-tepui in Venezuela (N5°09’50” W60°45’33”, 2,625 m). Roraima lies in the Pantepui biogeographical region and is one of the highest tepuis with a maximum elevation of 2,810 m and a summit area of ca. 35 km^2^^21^. The summit landscape is characterised by pioneering vegetation growing on acidic, oligotrophic sandstone soils with a mosaic of low-growing tepui forests, tepui scrubs, and high-mountain meadows and grasslands^22^. Although the summit experiences extreme environmental conditions with marked seasonal fluctuations^21,23^, sub-zero temperatures and frost have never been recorded. High hygrometry on the summit may prevent the air from reaching the freezing point^22^. Berry et al.^22^ described the summit climate as submicrothermic and ombrophilous, further characterised by high solar radiation, strong winds, heavy rainfall, persistent cloud and mist formation throughout most of the year, and a mean annual air temperature of 8–12 °C. Kok et al.^23^ reported a minimum air temperature of 6.5 °C, and a maximum air temperature of 20.4 °C, both recorded during the dry season; in the wet season they reported air temperature varying between 8.7 and 16.3 °C.

Only two amphibian species occur on the summit of Roraima-tepui^24^, both are direct developers (terrestrial eggs, no tadpole stage) and both species were used in most of our experiments:

*Oreophrynella quelchii* (Bufonidae) occurs in high numbers (millions of individuals) on the summit of Roraima-tepui. The species reaches ca. 20–29 mm body length and is only found on the summits of Roraima-tepui and Wei-Assipu-tepui, between ca. 2,200–2,800 m elevation in Venezuela, Guyana and Brazil. These terrestrial toads are active all-year round, both during the day and at night, and efficiently thermoregulate using thermally buffered microhabitats. While they are most commonly associated with vegetation, they demonstrate a broad ecological range by occupying almost all available summit microhabitats^23^.

*Pristimantis aureoventris* (Craugastoridae) reaches ca. 20–36 mm body length and is only found on the summits and upper slopes of Roraima-tepui, Wei-Assipu-tepui and Kukenán-tepui between ca. 2,200–2,800 m elevation in Venezuela, Guyana and Brazil. These frogs are rarely observed, with, to our knowledge, fewer than 50 known museum specimens. The species is active all-year round and is mostly nocturnal, although males also commonly call during the day. Unlike *O. quelchii*, their distribution on the summit is seemingly constrained by specific environmental parameters as they mostly inhabit bromeliads during the day and exhibit limited movement beyond moist environments, indicative of a higher sensitivity to water loss^24,25^ [PJRK, pers. obs.].

All experimental individuals were adults (40 *O. quelchii*, 4 *P. aureoventris*), collected by hand during the day (at air temperatures ranging between 12 and 16 °C) in the immediate vicinity of our field laboratory at the end of the dry season (March 2025). After collection, animals were kept at natural field temperatures (see above for seasonal ranges), and maintained under stable conditions of approximately 15 °C for 24 h before the experiments to standardise their physiological state. Animals were transported on foot and reached the field laboratory within 30 min of collection. All individuals were sexed in the field based on size, skin texture and/or vocalisations. Sexes were confirmed in the laboratory through dissection and examination of gonads. Sacrificed individuals were euthanised by immersion in 10 ml of 2% Linisol^®^ (lidocaïne hydrochloride; an amide class anaesthetic).

All experiments were carried out following relevant guidelines and regulations. Experimental protocols and the collection and handling of live animals were approved by the University of Lodz (Poland) and the Fundación para el Desarrollo de las Ciencias Físicas, Matemáticas y Naturales (Venezuela), and followed the guidelines outlined in the research permits (n° sitdb-hbuMVh8tAB and n° hzRrNCdmk) issued by the Ministerio Del Poder Popular Para El Ecosocialismo (MINEC, Venezuela) and the Instituto Nacional De Parques (INPARQUES, Venezuela).

### Measurements of critical thermal minima

The critical thermal minimum (CT_min_) is the lower T_b_ at which an animal loses its ability to function^26^. Prior to experiments 30 adult individuals of *O. quelchii* (10 males, 20 females) and four adult individuals of *P. aureoventris* (3 males, 1 female) were kept for 24 h at ca. 15 °C air temperature in small individual plastic boxes containing water-soaked paper towels to ensure proper hydration status. All individuals were weighed using an analytical balance A&D HR-100AZ (± 0.0001 g, values rounded to two decimal places) to control for biological variability. Experimental individuals were kept in a fasted state for 24 h to reduce gut content variability. By keeping animals in individual plastic boxes with water-soaked paper towels, we ensured similar hydration levels across individuals before cold exposure. The experimental individuals were (individually) gradually cooled at a controlled moderate-fast rate (-1 to -1.5 °C/min, an ecologically relevant and reproducible cooling rate; e.g.^27^, in an environmental chamber specifically designed for this study consisting of an Indel B TB15 OFF 15L-18 °C compressor cooling box equipped with two thermocouples (FLUKE 80PK-1, one at the top of the system, one close to the tested individual) attached to a HOBO^®^ 4-Channel Thermocouple Data Logger, and a HOBO^®^ MX2301 Temp/RH Data Logger to accurately record all environmental variables. The original top of the cooling box was replaced by a transparent PVC hard cover, with a small hole allowing the use of a telescopic pointer to flip the individual on its back for checking its righting response. Righting response was checked every 30 s, and CT_min_ was assumed to be reached when an individual could not right itself within 30 s after being flipped on its dorsum. A thermal image of the individual was immediately taken using a FLIR T540 thermal camera and the skin temperature (taken middorsal) was assumed to correspond to body temperature. We ran control tests to exclude any potential behavioral or fatigue effect; these tests included 10 control individuals that were similarly flipped to test their righting reflex over 20 min, but without experiencing temperature change. No individual lost its righting reflex during these control trials. We also ran validation tests to evaluate potential discrepancies between temperatures taken with an internal probe (FLUKE 80PK-1 inserted in the frogs/toads’ cloaca and attached to a HOBO^®^ 4-Channel Thermocouple Data Logger) vs. thermal images taken with the FLIR T540 thermal camera. Cloacal temperatures and thermal images were taken every 10 min for one hour at different temperatures to evaluate differences. No significant differences between the methods were found at the moderately-low tested temperatures (> -2 °C; although at lower temperatures we noticed up to ca. 2 °C differences, see below).

#### Statistical analyses

To evaluate whether critical thermal minima could be influenced by body weight or sex in *Oreophrynella* (sample size was too low for *Pristimantis*), we conducted two separate linear regression analyses with the critical thermal minimum (CT_min_, in °C) as the response variable. Body weight (g) was included as a continuous predictor, while sex was included as a categorical predictor. Model diagnostics were performed for each model to assess whether assumptions were met, Shapiro-Wilk test for normality of residuals, Breusch-Pagan test for homoscedasticity, and Durbin-Watson test for independence of residuals. Where heteroscedasticity was detected, robust standard errors (HC3) were applied. Additionally, a Welch’s two-sample t-test was performed to test for differences in body weight between sexes. The data set included 30 individuals (10 males, 20 females). All analyses were performed in R v4.5.1^28^ using base functions and the *lmtest*, *sandwich* and *car* packages for visualisation and diagnostics. Statistical significance was assessed at *p* < 0.05.

### Supercooling experiment and measurement of supercooling point

This experiment was designed to investigate the implications of the surprisingly low CT_min_ observed in *O. quelchii* (see Results). The same environmental chamber as described above was gradually cooled at a controlled moderate-fast rate (-1 to -1.5 °C/min) until air temperature next to the toad stabilised at ca. -8 °C. We deliberately restricted cooling to that temperature as it was (wrongly) expected to be lethal to the experimental animal. Skin temperatures were continuously recorded using a FLIR T540 thermal camera mounted on the cover of the chamber. Internal body temperatures were recorded using thermocouples (FLUKE 80PK-1) inserted in the toad’s cloaca and attached to a HOBO^®^ 4-Channel Thermocouple Data Logger. The tested individual (a female with a body weight of 1.51 g) was attached to a small styrofoam plate using a small piece of string secured around the thorax to avoid movements that could interfere with the stability of the cloacal temperature probe. In addition, we used a similarly equipped, fully hydrated plaster of Paris model as a control, placed next to the tested individual. These biophysical models are more durable than agar models and closely match agar models/living animals in water loss and temperature, while being easier to shape and colour^29^. Full temperature/hygrometry profiles were recorded in the environmental chamber during the experiment using data logger/thermocouple arrays. Cloacal thermocouples/data loggers recorded body temperature continuously. SCP was defined as the temperature at which an exothermic release (thermal spike) is observed (both on data loggers and thermal camera), indicating spontaneous freezing. In addition, the toad (and model) skin temperatures allowed precise detection of SCP as well as location of ice nucleation point through thermal imagery. This also informed us about differences between internal vs. skin temperatures. We noticed a difference of up to 2 °C between the cloacal probe and the thermal image at temperatures below ca. -5 °C, which we hypothesize could be due to thermal insulation/shielding, although we cannot formally rule out discrepancies in measurement equipment or calibration standards.

### Critical activity point (limit of dehydration tolerance)

This experiment aimed to explore a potential relationship between dehydration tolerance, CT_min_ and supercooling capacity.

#### Percentage of body water

To exclude the possibility of an atypically elevated body water content, we estimated the exact percentage of water in the body of *O. quelchii*. We selected five adult males and five adult females, which were kept fully hydrated (see above) and weighed using an analytical balance A&D HR-100AZ (± 0.0001 g, values rounded to two decimal places) before being euthanised by immersion in 10 ml of 2% Linisol^®^ (lidocaïne hydrochloride, an amide class anaesthetic). Specimens were immediately preserved in 70% ethanol for seven days, rinsed and transferred to a freezer at -24 °C for 14 days and freeze-dried (e.g.^30^) during 48 h using an Edwards Modulyo freeze dryer. Body water content was calculated as the difference between the fully hydrated body mass and the freeze-dried body mass, expressed as a percentage.

Due to the rarity of *P. aureoventris* and our small sample size (*n* = 4), we did not estimate exact body water percentage in this species.

#### Measurement of critical activity point

The critical activity point (CAP, a proxy used to estimate dehydration tolerance) is the amount of body (or water) loss that an individual can endure before being unable to right itself after being flipped on its dorsum^3,31^. CAP is, therefore, not the amount of water loss that causes death, but the amount of water loss that results in unresponsiveness to external stimuli; with all tested individuals expected to make a full recovery. Thirty fully hydrated adult individuals of *O. quelchii* (11 males, 19 females) and four fully hydrated adult individuals of *P. aureoventris* (3 males, 1 female) were kept at 15–17 °C T_b_ in individual boxes each containing a meshed sachet with 25–30 g of non-toxic amorphous silicon dioxide (SiO_2_) crystals. Individuals weight was recorded every 15 min until 20% body mass loss. Next, their weight was recorded every 10 min before flipping them on their dorsum. CAP was reached when the individual could not right itself within 30 s and expressed as a percentage of body mass. Dehydrated specimens were returned to individual containers with moist paper, and recovery time was monitored.

#### Statistical analyses

An independent samples t-test was conducted to compare body weights (g) between the two sexes in our dataset (*n* = 30; 11 males, 19 females) after assessing normality within groups using the Shapiro-Wilk test and homogeneity of variance between groups using Levene’s test. To explore the potential confounding effects of sex and body weight on dehydration tolerance (CAP, expressed as the percentage of body mass loss), we performed an ANCOVA including an interaction term between sex and weight. Because a significant interaction was detected, estimated marginal means (adjusted averages) were calculated to compare predicted CAP values between sexes at the overall mean body weight. In addition, to assess the overall relationship between CAP and body weight independently of sex, a simple linear regression was conducted using CAP as the response variable and body weight as the sole predictor. All analyses were performed in R v4.5.1^28^ using base functions and the *ggplot2*, *car*, *emmeans* and *patchwork* packages for visualisation and diagnostics. Statistical significance was assessed at *p* < 0.05.

Due to the rarity of *P. aureoventris* and our small sample size (*n* = 4), this species was not included in the statistical analysis.

### Skin bacterial communities profiling

This exploratory experimental approach was prompted by the unexpected observation that an *O. quelchii* skin swab stored in 2 ml of Nucleic Acid Preservation buffer (NAP) remained unfrozen after 5 days at -24 °C.

#### Swabbing

Swabbing was initially conducted as part of a separate study on the skin microbiota of *O. quelchii*. We used sterile swabs (Qiagen 4N6FLOQSwabs^®^) to swab the dorsum of 20 specimens of *Oreophrynella quelchii*, applying medium pressure for ca. 30 s^32^. These specimens were collected by hand, using sterile gloves, in the vicinity of our field laboratory and released at their capture site. Swabs were immediately placed in Nucleic Acid Preservation buffer (NAP, prepared following^33^, kept a few days at ca. 4–15 °C while still in the field, then frozen at -24 °C when back in the molecular lab.

#### DNA extraction

In addition to the swab that did not freeze (sample N05), we selected 4 swabs that froze (samples N01, N04, N07, N08, respectively; suggesting potential inter-individual variation in supercooling capacity) for sequencing. Microbial DNA was extracted using the Qiagen DNeasy PowerSoil Kit according to the manufacturer protocol. Extracted DNA was immediately sent to Genomed S.A. (Warsaw, Poland) for sequencing and bioinformatics.

#### Sequencing and bioinformatics

In brief, genomic DNA concentration was measured fluorometrically using the PicoGreen reagent prior to library preparation. Libraries were prepared using a two-step PCR with region-specific primers targeting the 16 S rRNA gene (V3–V4): 341 F (CCTACGGGNGGCWGCAG) and 785R (GACTACHVGGGTATCTAATCC)^34^. A first PCR was performed to amplify the target fragment and add adapter sequences. A second PCR was performed for amplification and addition of unique indices. Libraries were purified with magnetic beads after each PCR step. Concentration was measured again with PicoGreen, and sequencing was performed on the Aviti platform using paired-end technology. Demultiplexing and FASTQ generation were done using MiSeq Reporter v2.6 (Illumina). Taxonomic analyses were conducted in Qiime2 v2024.5^35^ using the SILVA 138.2^36^ reference database. Adapters and primers were removed with Cutadapt v4.7^37^. Quality trimming was done at Q30, and sequences shorter than 30 bp were discarded. Denoising was performed with DADA2^38^; via Qiime2). Paired reads were merged, unpaired and chimeric reads removed, and ASVs clustered at 99% similarity. ASVs were classified using a hybrid approach: (1) initial alignment with VSEARCH^39^; ≥50% identity, ≥ 80% coverage); (2) remaining ASVs classified using a Naive Bayes classifier (≥ 0.7 confidence).

## Results

### Critical thermal minima

*Oreophrynella quelchii* demonstrated a surprisingly low critical thermal minimum (CT_min_ as low as -1.90 °C, mean 0.46 °C ± 1.22, *n* = 30). Remarkably, the only other amphibian species reported from the summit of Roraima-tepui, the syntopic frog *P. aureoventris*, exhibited a much higher CT_min_ (lowest 4.90 °C, mean 6.05 °C ± 1.17, *n* = 4), which aligns more closely with current summit environmental conditions.

Linear models were used to test whether body weight or sex predicted the critical thermal minimum (CT_min_) of *O. quelchii*. Weight was not a significant predictor (Estimate = 0.26 ± 0.96, *p* = 0.788), with the model explaining only 0.3% of the variance (R² = 0.003). Similarly, sex showed no significant effect on CT_min_ (Estimate for males = -0.25 ± 0.49, *p* = 0.611), with low explanatory power (R² = 0.009). Residual diagnostics indicated that assumptions of normality (Shapiro-Wilk *p* > 0.63 for both models) and independence (Durbin-Watson *p* > 0.16) were met. However, the Breusch-Pagan test detected heteroscedasticity in the sex model (*p* = 0.017). To address this, heteroscedasticity-consistent (HC3) standard errors were applied, confirming the non-significant effect of sex (Estimate = -0.25 ± 0.39, *p* = 0.526). Aligning with the results of Kok et al.^23^, a Welch’s t-test confirmed a significant difference in body weight between sexes in our CT_min_ data set (*p* < 0.001), yet this did not translate into thermal performance differences (Figure S1A).

Due to the small sample size for *P. aureoventris* (*n* = 4), we did not perform statistical analyses for this species. Instead, descriptive values are reported to provide preliminary insight into its thermal tolerance (Table S1).

### Supercooling

**Fig. 1:**
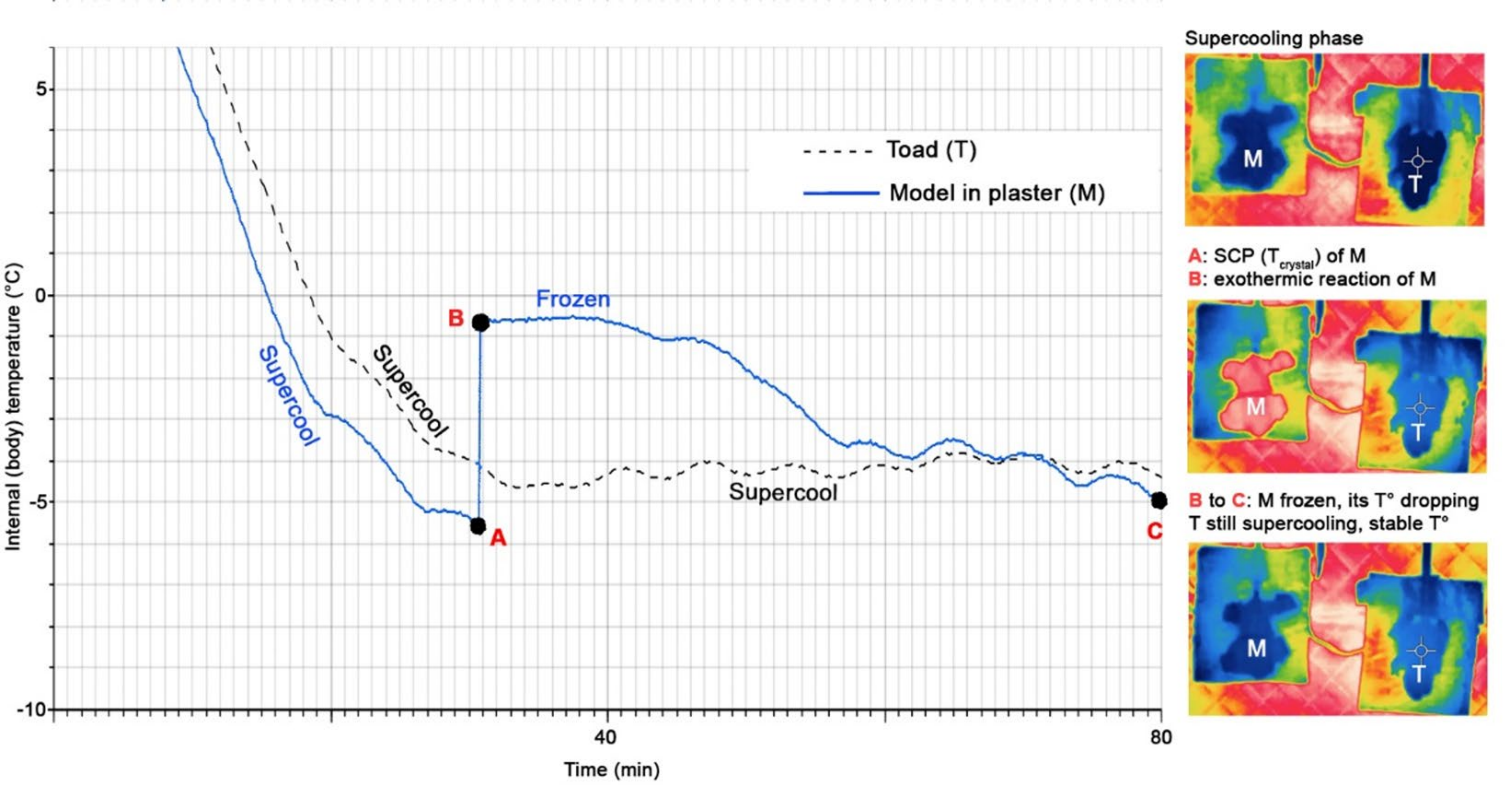
(Left) Cooling and freezing curves of a fully hydrated adult female *Oreophrynella quelchii* and a water-saturated plaster model (gypsum). Ice nucleation of the model started at **A** and freezing of the model continued from **B** to **C**. Note that the toad never reached SCP (i.e., never froze) and supercooled over the course of the experiment. (Right) Thermal images (from a video taken with a FLIR T540 thermal camera); note warmer colour (middle image) of plaster model during exothermic reaction, immediately after ice nucleation. The slight oscillations (< 0.5 °C) observed in both the toad and the plaster reflect passive temperature fluctuations within the chamber after it reached the set point, and should not be interpreted as an active physiological response. T_crystal_ = temperature of crystallisation = freezing/ice nucleation temperature.

Our tested female *O. quelchii* was capable of supercooling at least to -4.60 °C T_b_/-7 °C skin temperature (Fig. 1). This is among the lowest internal body temperatures recorded for a non-freezing amphibian, outperforming temperate species that routinely endure frost level temperatures. Since our experimental setup was never cooled beyond -8 °C air temperature near the toad (see Methods), the exact SCP of *O. quelchii* remains unknown but is likely below -4.60 °C.

The toad skin remained wet during all the experiment and the cloacal probe was easily removed at the end of the test. In contrast, due to freezing, the probe adhered to the plaster model, making removal challenging. Toad body weight loss was modest (ca. 10%). It is worth noting that the toad used in this experiment fully recovered from chill coma in ca. 1 h at 15 °C air temperature (returned to its plastic box containing water-soaked paper towel).

### Dehydration tolerance

Body water content in *O. quelchii* was estimated at 81.56% ± 1.26 (*n* = 5) in males and 79.20% ± 2.13 (*n* = 5) in females. When sexes are pooled, body water percentage is 80.38% ± 2.06 (*n* = 10), which aligns with available literature data on other anuran species (e.g.^40,41^), and do not suggest an atypically elevated body water content in *O. quelchii*. We relied on these results and literature data on other anuran species to estimate body water percentage in *P. aureoventris* at ca. 80%.

**Fig. 2:**
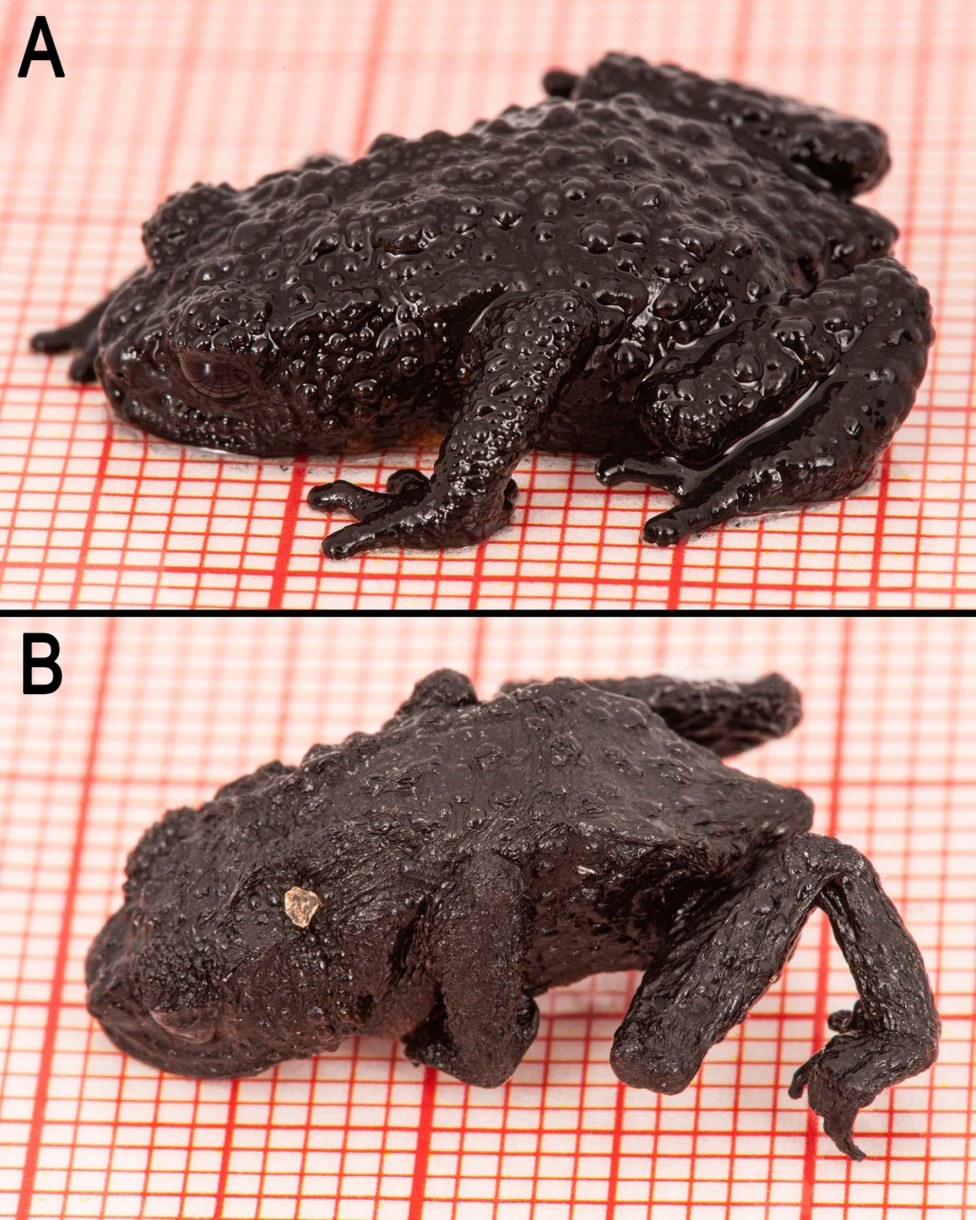
Male *Oreophrynella quelchii* before (**A**) and after (**B**) CAP estimation and loss of 36.5% body mass. Returned to a container with moist paper, this individual completely recovered—and fully regained its initial weight—in 4 h. A small silica (SiO_2_) crystal dislodged from the sachet and is visible on the dehydrated toad. Photos by P.J.R.K.

*Oreophrynella quelchii* exhibited a surprisingly high dehydration tolerance, with a CAP up to 47.7% body mass (37.55 ± 5.28, *n* = 30; up to 58.5% body water; Fig. 2). In contrast, *P. aureoventris* exhibited a much lower CAP, max 28.8% body mass (25.99 ± 1.96, *n* = 4; up to 35% body water). All tested specimens fully recovered in less than 24 h after the experiment, with no apparent adverse effects.

**Fig. 3:**
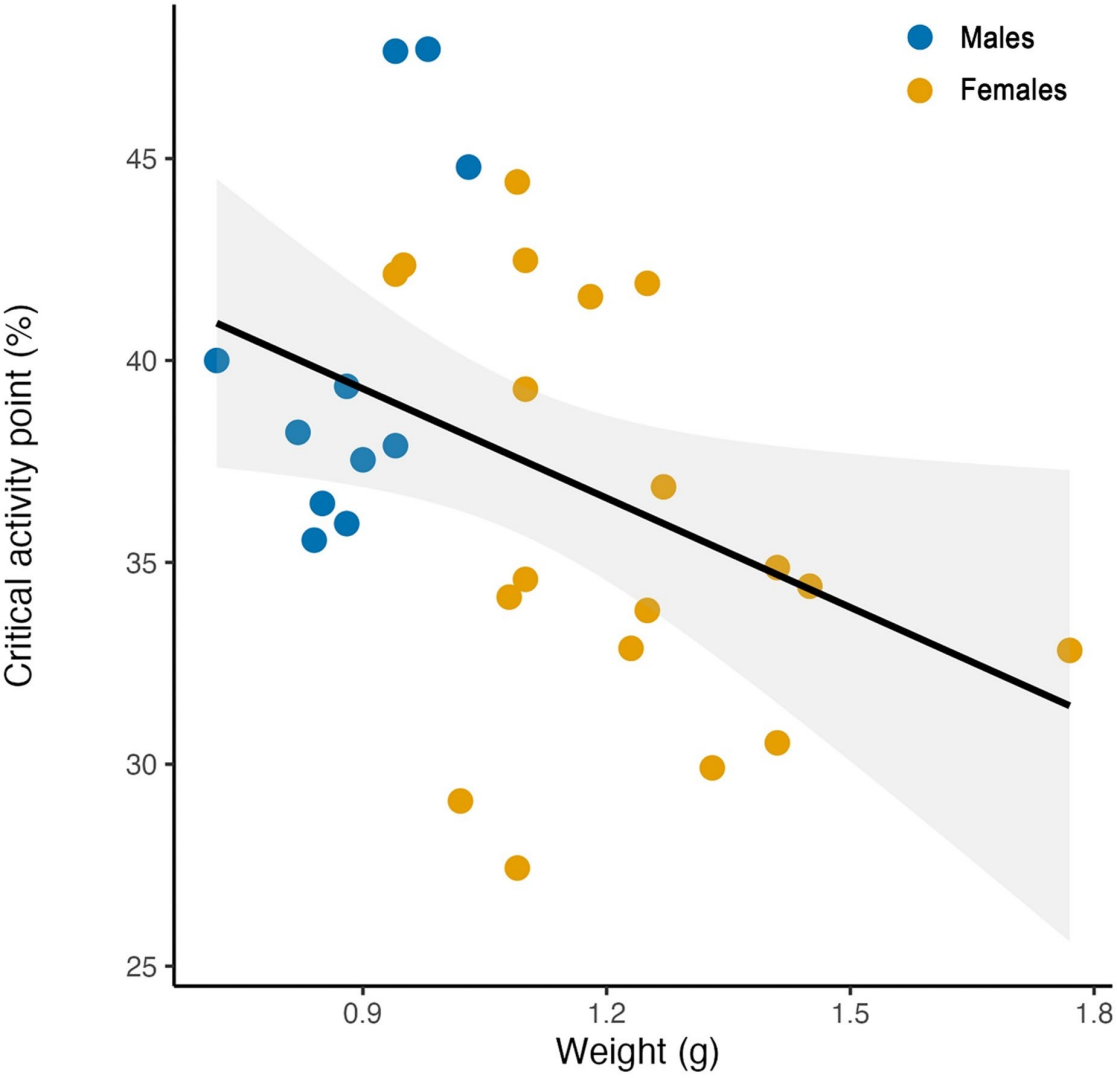
Scatter plot showing the relationship between CAP and body weight in *O. quelchii*, coloured by sex.

The Shapiro-Wilk test indicated no significant departure from normality in body weight within each sex (M = 0.980, *p* = 0.965; F = 0.913, *p* = 0.085). However, Levene’s test suggested heterogeneity of variance between sexes (F_1,28_ = 4.75, *p* = 0.038). Given this, a Welch’s t-test, which does not assume equal variances, was used to compare mean body weights between sexes. Females were significantly heavier than males in our CAP data set (t = 6.11, df ≈ 26.2, *p* < 0.001), consistent with previous findings (see above and^23^. An ANCOVA assessing dehydration tolerance (CAP%) as a function of sex and body weight revealed a significant interaction between sex and weight (F_1,26_ = 4.72, *p* = 0.039), indicating that the relationship between body weight and CAP differed between sexes (Fig. 3). At the overall mean body weight (1.11 g), estimated marginal means indicated higher CAP in males (46.3%, 95% CI: 38.4–54.2%) than in females (37.2%, 95% CI: 34.6–39.8%), although the overlapping confidence intervals suggest no significant difference at this weight level. Because of violation of homogeneity of variance and interaction effects, a simpler linear regression was conducted pooling both sexes, which showed that overall, dehydration tolerance decreased significantly with body weight (F_1,28_ = 5.10, *p* = 0.032; slope β = -9.03 ± 3.99 SE), explaining approximately 15% of variation in CAP.

Due to the small sample size for *P. aureoventris* (*n* = 4), we did not perform statistical analyses for this species. Instead, descriptive values are reported to provide preliminary insight into its dehydration tolerance (Table S2).

### Skin bacterial communities

**Fig. 4:**
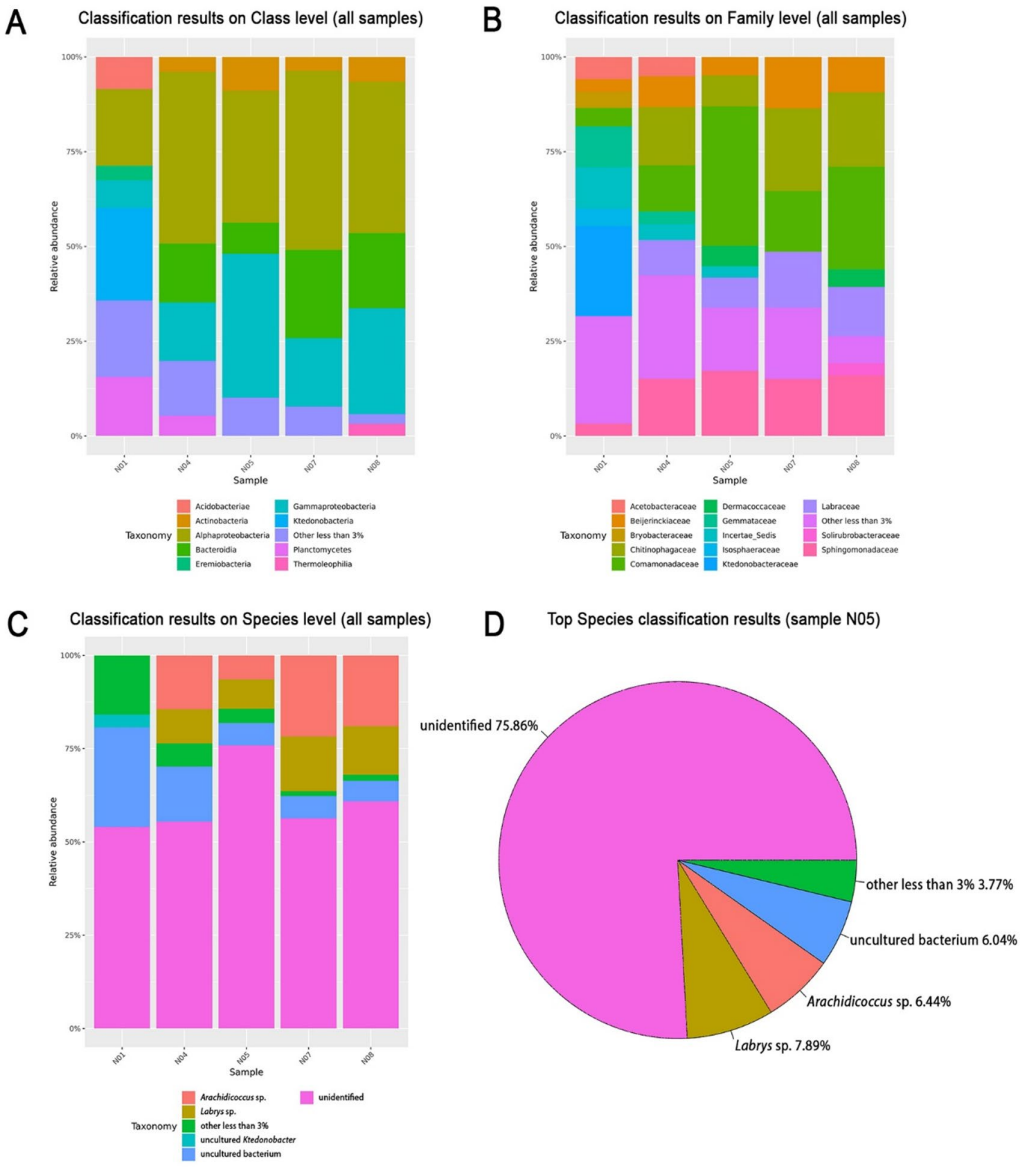
Taxonomic classification of skin bacterial communities from all *Oreophrynella quelchii* samples based on 16S rRNA sequencing. Bar plots show classification results at the (**A**) class, (**B**) family, and (**C**) species levels. A substantial proportion of sequences remain unclassified, especially at the species level. (**D**) Displays the top species-level classification results for sample N05, highlighting a dominant fraction of unclassified taxa (75.86%), followed by *Labrys* sp. (7.89%) and *Arachidicoccus* sp. (6.44%).

**Fig. 5:**
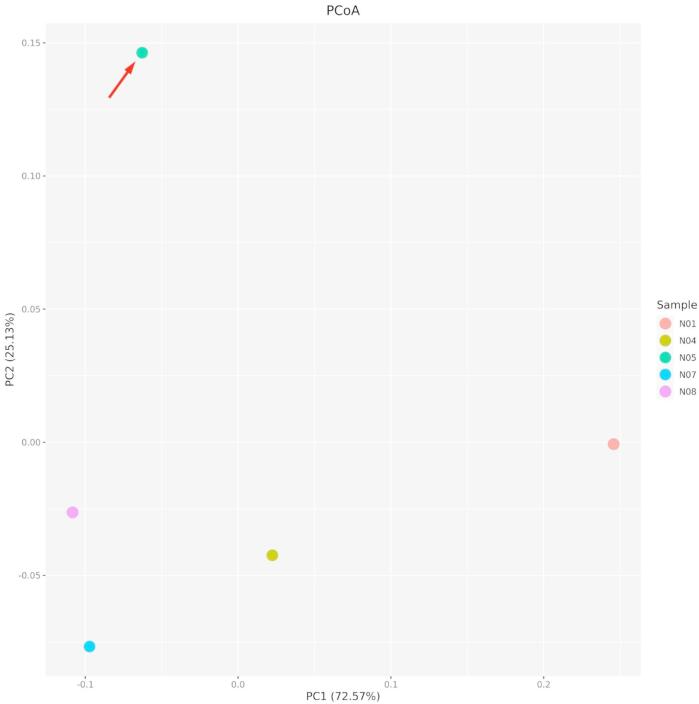
Principal coordinates analysis (PCoA) of skin bacterial community composition based on Bray-Curtis dissimilarity. Each point represents a skin microbiota sample (N01, N04, N05, N07, N08), with colours indicating individual samples. Axes PC1 and PC2 explain 72.57% and 25.13% of the total variance, respectively. The red arrow highlights sample N05 (a sample that did not freeze), which shows distinct microbial composition compared to other samples (which froze), particularly along PC2.

Over 75% of the bacterial species in the skin microbiota of sample N05 could not be confidently assigned to known species-level taxa (Fig. 4). However, a Principal Coordinates Analysis (PCoA, Fig. 5) based on Bray-Curtis dissimilarity shows sample N05 strongly separated from the other communities along PC1, which accounted for 72.6% of the variation, indicating a markedly distinct bacterial composition.

## Discussion

### Critical thermal minima and supercooling

Our results indicate that CT_min_ is not strongly influenced by sex or size-related traits in *O. quelchii*, suggesting that factors other than body weight and sex may be more critical in determining thermal tolerance limits in this species.

Negative or near zero values for critical thermal minima are rare in amphibians from latitudes below 20° (i.e., tropical), especially from areas that lack exposure to freezing temperatures (e.g.^42^).

We found a remarkable difference between the CT_min_ of *O. quelchii* (mean 0.46 °C) and *P. aureoventris* (mean 6.05 °C; aligning with current summit environmental conditions), although the species are syntopic and experience the same environmental conditions. Our findings indicate that *O. quelchii* is able to supercool at least to -4.60 °C T_b_/-7 °C skin temperature (Fig. 1), outperforming temperate species that routinely endure sub-zero temperatures. *Oreophrynella* being an ancient (> 30 million years^43^ local endemic and *Pristimantis* being a more recent (and widespread) tepui summit colonizer^44^, we interpret this as evidence that supercooling capacity in *O. quelchii* likely represents a retained ancestral adaptation that has persisted despite the absence of strong contemporary selective pressure. This supports the hypothesis of a Pantepui ancestor that evolved in situ and persisted through climatic oscillations.

In its natural environment, *O. quelchii* experiences pronounced circadian thermal oscillations, with air temperatures rarely dropping below ca. 7 °C, and individuals often occupying thermally buffered microhabitats. These daily cycles of cold exposure, likely historically more frequent and pronounced on tepui summits (see above), probably represent the primary selective pressure shaping thermal physiology in *Oreophrynella*, rather than seasonal variation as in temperate species. This context also supports our experimental design, which focused on short-term (< 2 h) exposure that mimic acute daily thermal challenges, testing supercooling potential under conditions that do not currently occur in the field.

Although intraspecific variability in supercooling capacity is unknown in *O. quelchii*, it is unlikely that the observed ability is unique to the tested individual, as it would be extremely improbable that only one randomly chosen specimen exhibits such pronounced supercooling.

In experimental conditions, *O. quelchii* cooled down more slowly and reached a higher stable temperature compared to a water-saturated plaster model that froze while subjected to identical conditions (Fig. 1). Although similar in size and water-saturated, the plaster model contained only ca. 20% water compared to ca. 80% in the toad. As freezing primarily depends on water availability, the toad’s higher water content should have made it more prone to ice nucleation and freezing under identical conditions. These findings strongly suggest that *O. quelchii* rely on supercooling-based mechanisms to delay ice formation. Since our experimental setup was never cooled beyond -8 °C air temperature near the toad, the exact SCP of *O. quelchii* remains unknown.

### Dehydration tolerance

*Oreophrynella quelchii* exhibited elevated dehydration tolerance, up to 47.7% of body mass, which corresponds to the upper physiological limit tolerated by most terrestrial anurans^3,20^, including species adapted to extreme water scarcity.

Our results suggest that dehydration tolerance decreases with body weight in *O. quelchii*, and that the slightly higher CAP observed in males can be explained by their smaller size. Prior studies have shown higher tolerance to dehydration in small amphibians, irrespective of the species (e.g.^45^). The observation that males of *O. quelchii* (which, on average, are lighter and smaller than females, see^23^ have a slightly higher CAP than females (Fig. 3) aligns with this size-related pattern. Our results also align with earlier reports suggesting that sexual differences in overall water metabolism are generally absent in amphibians (e.g.^45,46^). Our analyses indicate that body water content in males is marginally higher than in females (81.6% vs. 79.2%, see Results).

The modest body mass loss after supercooling (ca. 10%) rules out cryoprotective dehydration as a strategy^47^. However, elevated dehydration tolerance still contribute to the observed low CT_min_ and supercooling capacity.

In contrast, *P. aureoventris* exhibited low dehydration tolerance, with max 28.8% body mass loss. This supports our observations that *P. aureoventris* is ecologically constrained by specific moisture conditions and displays minimal dispersal outside these humid microhabitats, except at night when hygrometry is elevated.

### Potential role of amphibians skin microbiota in supercooling

Amphibians possess a moist, highly permeable integument with limited supercooling capacity compared to other tissues, excluding the gut^6^. Although freeze initiation has been linked with ice nucleation mediated by bacteria found on freeze-tolerant frog skin (e.g., *Pseudomonas*; see for instance^48–50^), to the best of our knowledge, there is no published study reporting the presence of bacteria producing anti-freeze proteins (AFPs) on the skin of amphibians. While certain bacteria are known to produce AFPs in cold environments (e.g.^51–55^), none of these have been identified as part of the amphibian skin microbiome.

The observation that not all *O. quelchii* skin swabs remained unfrozen is intriguing and suggests inter-individual variation in supercooling capacity. Similarly, the persistence of skin moisture at -8 °C air temperature is unexpected. As mentioned above, the skin, along with the intestinal tract, is reported to supercool the least in amphibians (i.e., freeze faster^6^. Over 75% of bacterial taxa in sample N05’s skin microbiota could not be assigned to known species, and PCoA (Fig. 5) revealed a distinct bacterial community, with N05 clearly separated along PC1. The proportion of unidentified bacterial species may represent novel lineages with yet uncharacterized functional roles, including potential contributions in modulating ice formation. Research on amphibian skin microbiota, a fast-growing field of biology, has primarily focused on aspects such as disease resistance and environmental interactions (e.g.^56–59^), with limited or no exploration into their role in cold tolerance or avoidance strategies. Our preliminary, circumstantial evidence suggests, for the first time, a potential contribution of skin-associated bacteria to amphibian freeze avoidance, which represents a promising avenue for future research.

## Conclusions

Our results indicate that amphibian supercooling capacity may be latent and not limited to freezing thermal environments. Physiological freeze avoidance in tropical montane amphibians is shown to be associated with low CT_min_, high dehydration tolerance and possibly antifreeze-producing skin microbiota, highlighting key physiological adaptations. These traits may determine species persistence under shifting climatic regimes, particularly in thermally variable montane systems. Our findings support the idea that selection for cold and drought tolerance may have driven local adaptation and enabled the long-term persistence of endemic lineages in Pantepui. A limitation of our study is that only a single specimen was tested for supercooling capacity. However, given the improbability that such pronounced supercooling occurs in only one randomly chosen individual, and considering that all other physiological tests were performed on at least 30 specimens, we consider the observed capacity likely representative of the species, while acknowledging that further testing would be needed to assess intraspecific variability. Understanding thermal limits in tropical ectotherms is critical, as even minor temperature shifts may exceed physiological thresholds. Future work should not only investigate whether these thermal traits are plastic or fixed across elevational gradients but also elucidate the molecular and physiological mechanisms underlying supercooling.

## Supplementary Information

Below is the link to the electronic supplementary material.

Supplementary Material 1

## Data Availability

Methods, including protocols for physiological assays and bioinformatics pipelines, are provided in the main text. Data and R codes used in this study are available on Figshare at 10.6084/m9.figshare.29777243. Raw data are also available in the electronic supplementary material. Raw metagenomic sequences generated and analysed during the current study are available in the NCBI SRA database at http://www.ncbi.nlm.nih.gov/bioproject/1303618, with the following accession numbers SAMN50518711, SAMN50518712, SAMN50518713, SAMN50518714, SAMN50518715.
