# Supplementary material for: Amphibian supercooling capacity is not limited to sub-zero thermal environments
Source: Sci Rep. 2025 Nov 17;15:40311. doi: 10.1038/s41598-025-24105-5 (PMC12623792; doi:10.1038/s41598-025-24105-5)
Supplement: Supplementary file 1 — Supplementary Material 1 [file 41598_2025_24105_MOESM1_ESM.pdf]

Electronic supplementary material

Amphibian supercooling capacity is not limited to sub-zero thermal environments

Philippe J. R. Kok, Bruno B. Wisse, Marlena Kapuściak, and Margarita Lampo

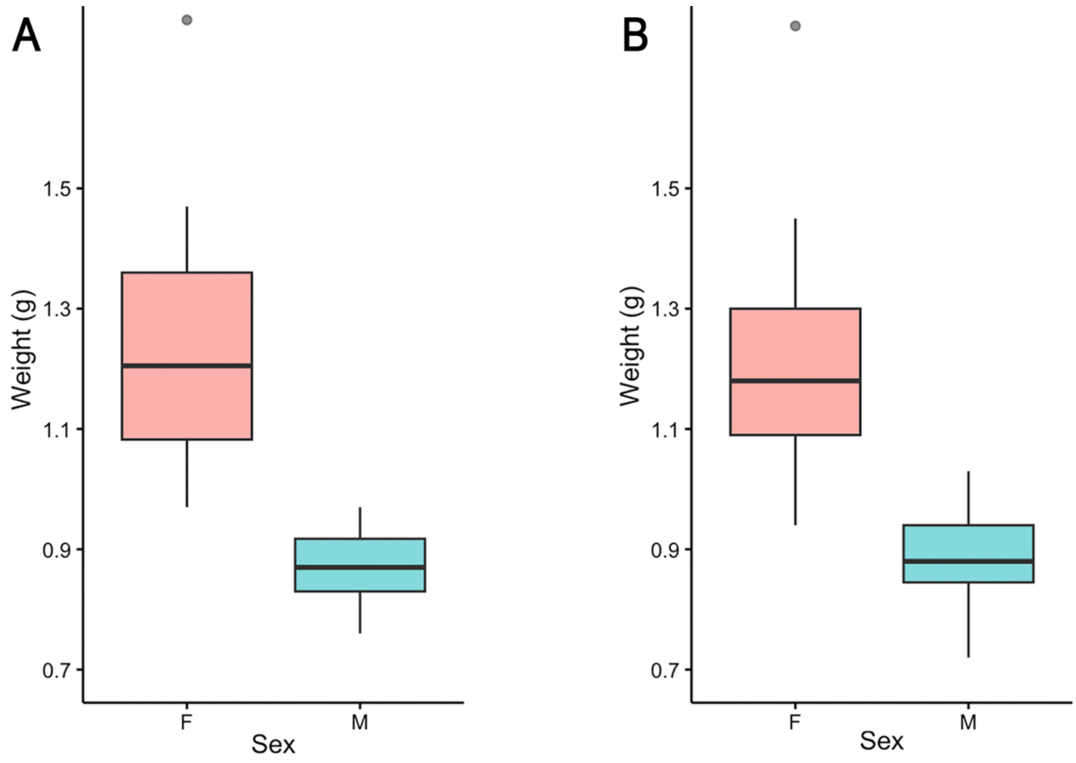

Figure S1. Body weight (g) by sex in our two data sets. (A) CT_{min} data set. (B) CAP data set. Boxplots display the distribution of body weight for *Oreophrynella quelchii* females and males. Boxes represent the interquartile range (IQR), horizontal lines indicate the median, and whiskers extend to 1.5×IQR.

Table S1. Estimated values of the critical thermal minima (CT_{min}) for the species and specimens used in this study. F = female; M = male. Weights were taken immediately before the experiments.

**Species**	**ID**	**Sex**	**Weight (g)**	**Ct_{min} (°C)**
Oreophrynella quelchii	O_002	F	1.14	1.6
Oreophrynella quelchii	O_003	F	0.99	-0.7
Oreophrynella quelchii	O_004	M	0.86	0.0
Oreophrynella quelchii	O_005	F	1.39	1.5
Oreophrynella quelchii	O_006	M	0.88	0.9
Oreophrynella quelchii	O_007	F	1.11	-1.5
Oreophrynella quelchii	O_008	F	1.09	-1.7
Oreophrynella quelchii	O_009	F	1.17	0.3
Oreophrynella quelchii	O_010	F	1.16	0.8
Oreophrynella quelchii	O_011	F	1.78	2.7
Oreophrynella quelchii	O_012	F	1.35	0.1
Oreophrynella quelchii	O_013	F	1.30	-0.9
Oreophrynella quelchii	O_015	F	1.47	-1.9
Oreophrynella quelchii	O_016	F	1.31	1.4
Oreophrynella quelchii	O_017	M	0.92	0.2
Oreophrynella quelchii	O_018	F	1.00	3.0
Oreophrynella quelchii	O_020	M	0.86	1.2
Oreophrynella quelchii	O_021	F	1.24	0.3
Oreophrynella quelchii	O_022	M	0.91	-0.1
Oreophrynella quelchii	O_023	F	1.46	-0.7
Oreophrynella quelchii	O_024	M	0.76	-0.1
Oreophrynella quelchii	O_025	M	0.80	0.3
Oreophrynella quelchii	O_026	F	1.02	0.4
Oreophrynella quelchii	O_027	M	0.96	-0.5
Oreophrynella quelchii	O_028	M	0.82	-0.1
Oreophrynella quelchii	O_029	M	0.97	1.1
Oreophrynella quelchii	O_030	F	1.06	2.5
Oreophrynella quelchii	O_031	F	1.43	0.3
Oreophrynella quelchii	O_032	F	0.97	2.2
Oreophrynella quelchii	O_034	F	1.24	1.1
Pristimantis aureoventris	P_001	M	1.98	4.9
Pristimantis aureoventris	P_002	M	1.85	5.2
Pristimantis aureoventris	P_003	M	1.71	6.2
Pristimantis aureoventris	P_004	F	3.58	7.9

Table S2. Estimated values of the critical activity points (CAP, expressed in % body mass) for the species and specimens used in this study (see main text for details).

Species	ID	Sex	Weight before (g)	Weight after (g)	CAP
Oreophrynella quelchii	O_004	M	0.88	0.56	35.96
Oreophrynella quelchii	O_005	F	1.10	0.67	39.29
Oreophrynella quelchii	O_006	M	0.94	0.49	47.66
Oreophrynella quelchii	O_007	F	1.10	0.72	34.58
Oreophrynella quelchii	O_009	F	1.09	0.79	27.43
Oreophrynella quelchii	O_011	F	1.77	1.19	32.82
Oreophrynella quelchii	O_013	F	1.25	0.83	33.81
Oreophrynella quelchii	O_018	F	1.02	0.72	29.09
Oreophrynella quelchii	O_023	F	1.45	0.95	34.41
Oreophrynella quelchii	O_024	M	0.82	0.51	38.22
Oreophrynella quelchii	O_025	M	0.90	0.56	37.54
Oreophrynella quelchii	O_026	F	0.95	0.55	42.36
Oreophrynella quelchii	O_027	M	0.85	0.54	36.46
Oreophrynella quelchii	O_028	M	0.94	0.58	37.89
Oreophrynella quelchii	O_029	M	0.98	0.51	47.71
Oreophrynella quelchii	O_030	F	1.25	0.73	41.91
Oreophrynella quelchii	O_031	F	1.41	0.98	30.53
Oreophrynella quelchii	O_032	F	1.10	0.64	42.48
Oreophrynella quelchii	O_033	F	0.94	0.55	42.14
Oreophrynella quelchii	O_034	F	1.27	0.80	36.87
Oreophrynella quelchii	O_035	M	1.03	0.57	44.79
Oreophrynella quelchii	O_036	F	1.09	0.60	44.42
Oreophrynella quelchii	O_037	F	1.41	0.92	34.87
Oreophrynella quelchii	O_038	M	0.84	0.54	35.55
Oreophrynella quelchii	O_039	F	1.23	0.83	32.87
Oreophrynella quelchii	O_040	F	1.33	0.94	29.91
Oreophrynella quelchii	O_041	M	0.72	0.43	40.00
Oreophrynella quelchii	O_042	M	0.88	0.53	39.36
Oreophrynella quelchii	O_043	F	1.08	0.71	34.14
Oreophrynella quelchii	O_046	F	1.18	0.69	41.58
Pristimantis aureoventris	P_001	M	2.17	1.62	25.30
Pristimantis aureoventris	P_002	M	1.67	1.24	25.76
Pristimantis aureoventris	P_003	M	1.75	1.24	28.75
Pristimantis aureoventris	P_004	F	3.75	2.84	24.15
